# Isolation and Characterization of the *Etheostoma tallapoosae* (Teleostei: Percidae) CENP-A Gene

**DOI:** 10.3390/genes2040829

**Published:** 2011-10-31

**Authors:** Dyanna M. Fountain, Leos G. Kral

**Affiliations:** Department of Biology, University of West Georgia, Carrollton, GA 30118, USA; E-Mail: dfountain82@gmail.com

**Keywords:** CENP-A, gene structure, *Etheostoma tallapoosae*, PCR based gene walking

## Abstract

Both centromeric alpha-satellite sequences as well as centromeric protein A (CENP-A) are highly variable in eukaryotes. CENP-A, a histone H3 variant, is thought to act as the epigenetic “mark” for assembly of centromeric proteins. While most of the histone fold domain (HFD) of the CENP-A is fairly well conserved, a portion of this HFD as well as the N-terminal tail show adaptive variation in both plants and animals. Such variation may establish reproductive barriers that may lead to speciation. The family Percidae contains over 200 species most of which are within the subfamily Etheostomatinae. This subfamily represents a species rich radiation of freshwater fishes in North America and these species exhibit both allopatric and sympatric distributions. In order to study the evolution of CENP-A in percid fish species, we have isolated and characterized the CENP-A gene from *Etheostoma tallapoosae* by PCR based gene walking. As a result of this study we have demonstrated that the Tallapoosa darter CENP-A gene HFD sequences can be isolated from genomic DNA by nested PCR in a manner that does not lead to the amplification of the highly sequence related histone H3 gene. We also demonstrated that PCR based walking can be subsequently used to isolate the rest of the CENP-A gene and adjacent gene sequences. These adjacent gene sequences provide us with a primer binding sites for PCR isolation of the CENP-A gene from other percid species of fishes. An initial comparison of three percid species shows that the N-terminal tail of the percid CENP-A gene shows adaptive evolution.

## 1. Introduction

Centromeric protein A (CENP-A) is a histone H3 variant that is thought to act as the epigenetic “mark” for the assembly of all centromertic proteins (see reviews [1,2]). While centromeric regions in most animals and plants contain repetitive α-satellite DNA segments [3], neocentromers can form in absence of these repetitive sequences [4]. Deposition of CENP-A thus appears to be DNA sequence independent. Furthermore, in human and yeast cells, only loop 1 (L1) and α helix 2 of the histone fold domain (HFD) of CENP-A specify the localization of that protein to the centromere. This region is termed the centromere targeting domain (CATD). Specifically, when loop 1 and alpha helix 2 of histone H3 are replaced with the CATD from CENP-A, the H3-CATD chimeric protein not only localizes to centromeres but also functionally rescues cultured cells depleted of endogenous CENP-A [5]. The CATD appears to confer centromere specificity by giving rise to a more rigid nucleosomal structure [6,7].

Interestingly, various regions of CENP-A (and of its orthologs CenH3 in plants and Cid in *Drosophila*), show adaptive variation in a variety of organisms. In *Drosophila*, adaptive evolution of Cid was detected in both the N-terminal tail as well as in the HFD with most of the adaptive changes being localized in L1 of the CATD [8]. Indeed, the L1 region was shown to confer species specific targeting of chimeric Cid to centromeres [9].

Adaptive variation of CenH3 was initially detected only in the N-terminal tail in a comparison of two *Arabidopsis* species [10]. Adaptive variation was then also detected in the L1 of the CATD in an expanded analysis of CenH3 from several species in the Brassicaceae family [11]. Functionality of various CenH3 domains was determined by testing various CenH3-H3 chimeras in an *Arabidopsis thaliana* null mutant [12]. Interestingly, the H3-CATD chimera was unable to complement the lethal null mutation. A chimeric protein containing the entire CenH3 histone fold domain and the H3 N-terminal tail restored viability (enabled mitosis) to the null mutant but the transfromants were sterile. Both the CenH3 N-terminal tail as well as the CenH3 HFD, were necessary for meiosis. That adaptive variation of CenH3 observed among species of the Bressicaceae family is of functional significance was demonstrated by the observation that a CenH3 transgene from only a closely related species was able to functionally complement the *A. thaliana* null mutation [12].

An initial analysis of CENP-A from a sampling of mammalian species failed to detect any adaptive variation [13]. A comparison of mouse, rat, and Chinese hamster CENP-A sequences only revealed purifying selection in those rodent lineages. Lack of adaptive variation was also found in the comparison of human, chimpanzee and bovine CENP-A sequences. However, a recent study that compared the CENP-A sequence in 14 species of primates (several species each of apes, Old World monkeys, New World monkeys and prosimians) detected adaptive variation in both the N-terminal tail as well as the HFD [14]. The adaptive variation observed in the HFD did not extend to the CATD with the exception of one amino acid change observed between prosimian and the other primate sequences at the start of the CATD.

At this time, adaptive variation of CENP-A has not been studied in other vertebrates. In this study we have isolated and characterized the CENP-A gene and portions of adjacent genes from *Ethesostoma tallapoosae*, the Tallapoosa darter. The adjacent gene sequences were utilized to design primers for the PCR amplification of this gene from two *Percina* species to obtain sequences for the comparative analysis of percid CENP-A evolution.

## 2. Results and Discussion

### 2.1. Isolation of the Tallapoosa Darter CENP-A Gene

The amino acid sequence of CENP-A shares a great deal of identity to that of histone H3 within the HFD. The N-terminal tail is highly diverged not only between these two proteins but also between CENP-As of different species. Our strategy for isolating the Tallapoosa darter CENP-A gene was to initially design PCR primers based on the conserved fish CENP-A HFD sequences that are partly divergent between fish CENP-A and histone H3. These primers were then used to amplify a portion of the darter CENP-A HFD. From this partial HFD sequence, Tallapoosa darter specific primers were then designed for PCR based walking both upstream and downstream to isolate the rest of the gene.

**Figure 1:**
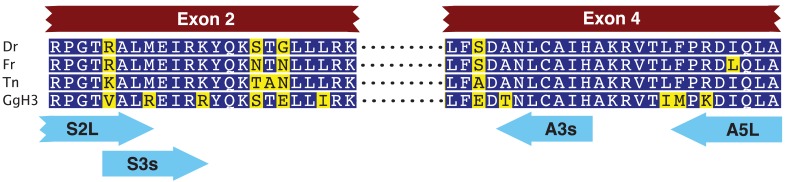
Locations of nested PCR primers that specifically amplify a portion of the fish CENP-A gene and not the histone H3 gene. Arrowheads indicate the 3′ ends anchored in codons that differentiate CENP-A from H3. CENP-A sequences: Dr: *Danio rerio*, Fr: *Takifugu rubripes*, Tn: *Tetraodon nigrovirdis*. Vertebrate histone H3: GgH3: *Gallus gallus* histone H3. Alignment based on Regnier *et al.* [15].

We first designed two pairs of nested primers such that the 3′ ends of these primers were anchored in codons that code for amino acids that differ within the HFD between fish CENP-A and vertebrate histone H3 (Figure 1, Table 1) [15]. With these primers we amplified a 3,162 nucleotide long fragment that spanned the CENP-A gene from within exon 2 to within exon 4. Exon-intron junctions were deduced from open reading frames that matched fish CENP-A sequences. We then designed gene specific nested primers just downstream of exon 2 (F2(AP1) and F2(AP2)) and just upstream of exon 4 (BC(AP1) and BB(AP2)) that were used in PCR based walking reactions (Table 2). The initial upstream walk only extended the CENP-A gene 315 nucleotides upstream of exon 2. Another set of nested walking primers was designed upstream of exon 2 (W2C(AP1) and W2A(Ap2)) (Table 2). The second upstream walk generated a DNA sequence that encompassed all of exon 1 and extended 2,731 nucleotides upstream of the CENP-A initiation codon. This upstream sequence encodes exon 1 of the EAF1 gene that codes for ELL associated factor 1, one of the positive regulators of RNA polymerase II elongation factor ELL [16]. The initial downstream walk extended the darter CENP-A gene sequence 115 nucleotides downstream of the stop codon that is present in exon 4. Utilizing additional restriction digested DNA libraries, the repeated downstream walk extended 1,934 nucleotides downstream of exon 4 and this sequence encodes exon 1 of methyltransferase-like protein 6.

**Figure 2:**
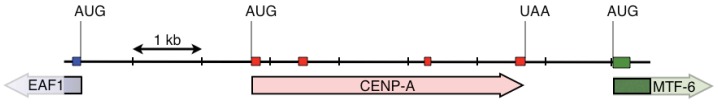
Schematic representation of the Tallapoosa darter CENP-A gene structure, the upstream location of exon 1 of the EAF1 gene and the downstream location of the methyltransferase-like protein 6 gene.

CENP-A gene and the neighboring portions of the EAF1 and the methyltransferase-like protein 6 genes are shown in Figure 2. The CENP-A gene is encoded within 4 exons. This gene structure is the same as in *Gasterosteus aculeatus* (three-spined stickleback), *Takifugu rubripes* (fugu pufferfish), and *Tetraodon nigrovirdis* (spotted green pufferfish). Interestingly, the CENP-A gene in *Danio rerio* (zebrafish) lacks introns. The structures of these genes were obtained from blastp searches of Ensembl genomic databases [17]. It should be noted that the CENP-A gene is not between to the EAF1 and the methyltransferase-like protein 6 genes in any of these other fish species as determined by examination of the annotated genomic sequences in the various genomic databases.

### 2.2. Comparative Sequence Analysis of Fish CENP-A

**Figure 3:**
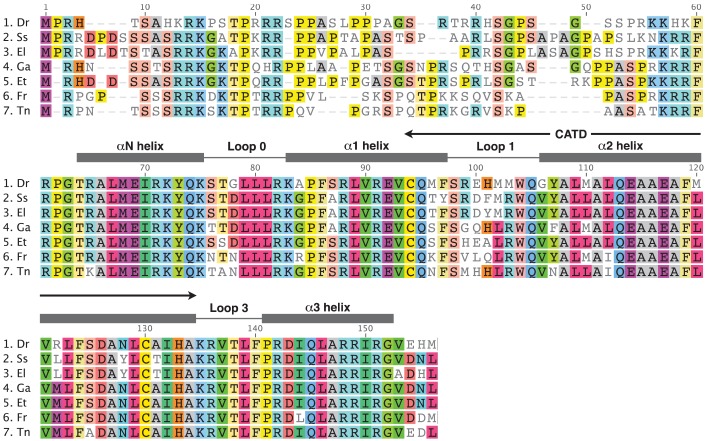
Alignment of deduced amino acid sequences of the fish CENP-A protein. Structural and functional features of the HFD are indicated above the alignment according to Regnier *et al.* [15] and Schueler *et al.* [14]. Dr: *Danio rerio*, Ss: *Salmo salar*, El: *Esox lucius*, Ga: *Gasterosteus aculeatus*, Et: *Ethesostoma tallapoosae*, Fr: *Takifugu rubripes*, Tn: *Tetraodon nigrovirdis*.

In order to carry out a comparison of CENP-A amino acid sequences between the Tallapoosa darter and all other fish species for which the CENP-A sequence has been obtained, we carried out blastp and tblastn searches of GenBank databases with the Tallapoosa darter CENP-A HFD. In addition to the zebrafish and pufferfish sequences previously identified in the databases by Regnier *et al.* [15], we also identified cDNA sequences from *Salmo salar* (salmon) and *Esox lucius* (northern pike). An alignment of all these fish CENP-A sequences is shown in Figure 3. The high degree of homology between the Tallapoosa darter sequence and all the other fish CENP-A sequences confirms that the Tallapoosa darter CENP-A gene has been isolated.

**Table 1:** Nested primer pairs used to amplify Tallapoosa darter CENP-A gene from within exon 2 to within exon 4.

**Primer Name**	**Primer Sequence (5′ to 3′)**	**Annealing Temperature (°C)**
S2L	TCGNCCNGGRACNARGGCCCTRATG	66
A5L	TGGCCARCTGNANGTCRCGNGGRAA	66

S3s	GGCCCTRATGGARATYCGCAA	64
A3s	ATGGATGGCACANARRTTNGC	64

**Table 2:** Nested primers for PCR based walking reactions.

**Primer Name**	**Primer Sequence (5′ to 3′)**
F2(AP1)	CAGAAAACATGCCGAGTCTTACTCCTG
F1(AP2)	CTCCTCGTGGTTTGCTTCTGTTTGACT

BC(AP1)	CAAGTTATAGAAGCTGCATTTATTGTTTTC
BB(AP2)	GTGCCTGGTGATGTTTAGTTTTGTAAC

W2C(AP1)	TCTGACAGAAAGTGTTCGCTCCCAGAC
W2A(AP2)	CAGTAGGAGTCCCATGTGCAATAAATCGG

As in other groups of organisms [8,11,13-15], the most variable regions in fish CENP-A are in the N-terminal domain and in the L1 of the HFD. The HFD as a whole is highly conserved showing and average 86.1% identity in pair wise comparisons. With the exception of the two pufferfish species, all of the other fish species being compared are in different orders and these are grouped into three different superorders. The zebrafish is in the superorder Ostriophysi. The salmon and pike species are both in the superorder Protacanthopterygii and the stickleback, darter and pufferfish species all group into the superorder Acanthopterygii. The two pufferfish species are both in the Tetraodontidae family. Interestingly, a number of amino acid variations show lineage specific patterns which may indicate that these changes are maintained by purifying selection over long evolutionary time periods (Figure 3). For example, the following pattern differentiates the Acanthopterygii lineages from those of the other species. At alignment position 2 there is a deletion of a Pro between the conserved Met and Arg amino acids. At position 53 and 54 a conserved Ala-Ser pair is present. At position 102 Leu replaces Met, and at position 122 Met replaces Leu (salmon and pike) or Arg (zebrafish).

### 2.3. Comparison of CENP-A in Etheostoma and Percina Species

We employed the following strategy to PCR amplify the CENP-A gene from *P. austroperka* and *P. roanoka*. First, we utilized nested primer pairs EAFo-tdA5L and EAFi-tdA3s (Table 3) to amplify from the EAF-1 gene to within exon 4 of the CENP-A gene from each of the *Percina* species. The tdA5L and tdA3s primers are non-degenerates versions of primers A5L and A3s (Table 1) based on the Tallapoosa darter sequence. *Percina* amplifications failed with the degenerate primers. From the obtained amplimer sequences, species specific primers fwPA1 (for *P. austroperka*) and fwPR1 (for *P. roanoka*) (Table 3) were designed just upstream of exon 4 and all of exon 4 from these species was amplified using these primers and primer revMTF6 (Table 3). This reverse primer was designed to bind within the conserved region of the methyltransferase-like protein 6 exon 1. The entire *Percina* CENP-A gene sequences were the assembled from these two sets of sequences. While the entire CENP-A gene can be amplified in one step utilizing the EAFi and revMTF6 primer pair, the two step process described above was utilized since the EAFi–revMTF6 amplimer length significantly exceeds the 6,000 nucleotide limit of the Clontech Advantage 2 Polymerase PCR Kit used in this study.

**Table 3:** Primers utilized for amplification of CENP-A gene from *Percina* species.

**Primer Name**	**Primer Sequence (5′ to 3′)**	**Annealing Temperature (°C)**
EAFo	TCTCCGGGCTTCAAAACATGCTCCTC	63
tdA5L	TGGCCAGCTGAATGTCCCGAGGGAA	63

EAFi	GCGGATTCGTGCTCCCGTTCA	63
tdA3s	GTGGATCGCACACAGGTTCGC	63

fwPA1	TGCGTGATGAATACAGTGCCTGGT	63
fwPR1	CCGCGTCCCAAACACACCGA	63
revMTF6	TCCCTGGTGGTCCAGTGTCTGTC	63

**Figure 4:**
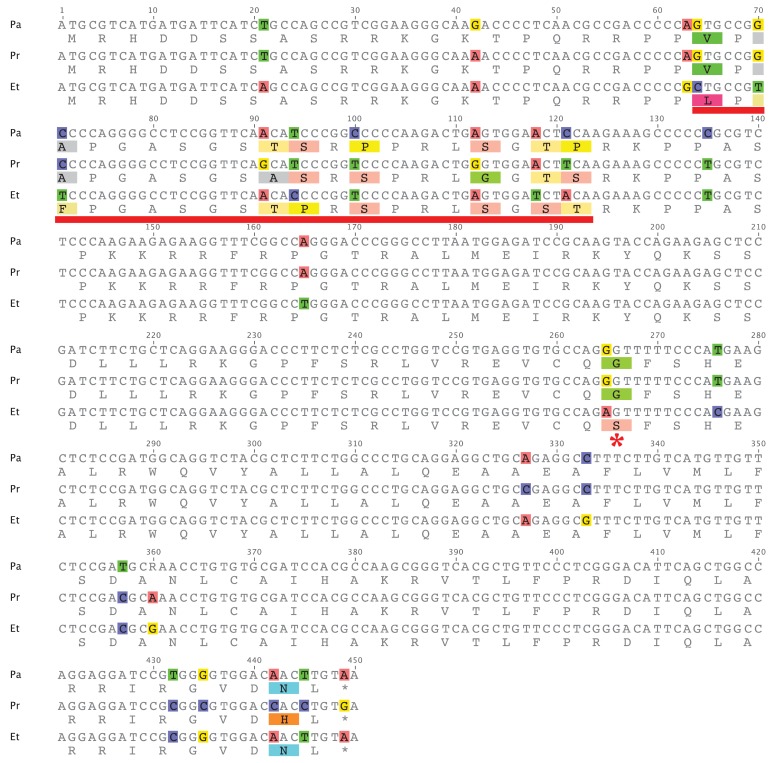
Comparative sequence analysis of CENP-A of *P. austroperka* (Pa), *P. roanoka* (Pr), and *E. tallapoosae* (Et). The red underline indicates a region within the N-terminal tail where Ka > Ks.

An alignment of the coding sequences from *E. tallapoosae*, *P. austroperka,* and *P. roanoka* is shown in Figure 4. To determine if evidence of positive selection could be obtained from this sequence comparison, pairs of these sequences were initially subject to sliding window analysis utilizing the SWAKK web server [18]. This initial analysis showed that a K_A_ > K_S_ signal was obtained in a 20 amino acid segment in the middle of the N-terminal tail (underlined in red in Figure 4). Statistical significance of this positive selection signal was tested by both the Z-test of Selection and by Fisher's Exact Test of Selection [19] where the numbers of synonymous and non-synonymous differences between sequences were estimated using the Nei-Gojobori method [20]. As indicated in Table 4 and Table 5, both of the tests show that in this portion of CENP-A the rate of non-synonymous substitutions is significantly greater than the rate of non-synonymous substitutions between *E. tallapoosae* and the two *Percina* species (p < 0.05). The remainder of the sequence is under purifying selection (data not shown).

This data shows that while most of the CENP-A gene in percids appears to be under purifying selection, at least a portion of the N-terminal tail is adaptively evolving as it is in the CENP-A of *Drosophila, Arabidopsis*, and primates. While adaptive variation was detected in the L1 region of the CATD in *Drosophila* and in members of the Brassicaceae family, no such variation was observed in the L1 region of the percids examined in this study. The *Etheostoma* and *Percina* lineages diverged about 20 to 35 million years ago [21]. As in similarly related primate taxa [14], the CATD sequences are nearly identical indicating a high degree of purifying selection affecting the L1 portion of the CATD. There is only one non-sysnonymous change within the α 1 helix portion of the CATD sequence where a Ser in *Etheostoma* is replaced with a Gly in *Percina* (Figure 4, indicated by *).

**Table 4:** Codon-based test of positive selection for analysis between percid CENP-A sequences (Pa: *P. austroperka*, Pr: *P. roanoka*, and Et: *E. tallapoosae*). The probability of rejecting the null hypothesis of strict-neutrality (dN = dS) in favor of the alternative hypothesis of positive selection (dN > dS) at the middle portion of the N-terminal tail (Figure 4, red underlined sequences) is given below the diagonal. Values of less than 0.05 are considered significant. The test statistic (dN – dS) is shown above the diagonal.

	**Pa**	**Pr**	**Et**
Pa		2.6911	2.5770
Pr	0.0125		2.8992
Et	0.0056	0.0022	

**Table 5:** Results from Fisher's exact test of neutrality for pairs of percid CENP-A sequences (Pa: *P. austroperka*, Pr: *P. roanoka*, and Et: *E. tallapoosae*). The probability (p) of rejecting the null hypothesis of strict-neutrality in favor of the alternative hypothesis of positive selection at the middle portion of the N-terminal tail (Figure 4, red underlined sequences) is shown for each pair. Values of p < 0.05 are considered significant.

	**Pa**	**Pr**	**Et**
Pa			
Pr	0.1687		
Et	0.0483	0.0301	

### 2.4. Significance

The evolution of CENP-A and other centromeric/kinetichore proteins is hypothesized to be in response to changes in centromeric satellite DNA elements that can result in “centromere drive” that can distort meiotic chromosomal segregation in the heterogametic sex [8,22,23]. The adaptive variations of centromeric components may give rise to functional incompatibilities during meioses of hybrids of diverged populations thus leading to reproductive isolation and subsequent speciation [8,22,23]. This study demonstrates that adaptive evolution of at least a portion of the CENP-A gene has occurred in the percid lineages examined. Further characterization of this adaptive variation of CENP-A in the other percid species may lead to insights about the evolutionary history of this highly diverse family of fishes.

## 3. Experimental

### 3.1. DNA Source Materials and DNA Isolation

Genomic DNA from *E. tallapoosae* had been obtained previously by Brogdon *et al.* [24]. Genomic DNA or tissue samples from *P. austroperka* and *P. roanoka* were kindly provided by Tom Near, Yale University. DNA was isolated from tissue samples with the Qiagen DNeasy Blood and Tissue Kit according to manufacturer's instructions.

### 3.2. PCR Primer Design

All PCR primers were designed with Primer Premier (Biosoft) or with Geneious Pro bioinformatics software (Biomatters Ltd.). Degenerate nested primers S5L, S3s, A5L and A3s were designed based on *T. nigrovirdis* CENP-A sequences by constraining the 3′ end of the primer locations as discussed in 2.1 above. Reasonable degeneracy was introduced into these primer sequences by comparing the primers to alignments of those exon 2 and exon 4 CENP-A DNA sequences from other fish sequences obtained from GenBank and Ensembl databases and to reverse translations of the relevant conserved fish amino acid sequences. Optimum annealing temperatures for the primer pairs were determined by temperature gradient PCR amplification with an Eppendorf Mastercycler.

Gene specific nested primers for PCR based walking were designed from obtained darter CENP-A sequences for compatibility with AP1 and AP2 primers provided as part of the GenomeWalker Kit (Clontech). The gene specific primers were constrained to be 26 to 30 nucleotides in length with a GC content of 40% to 60% with an annealing temperatures above 67 °C.

All other PCR primers were designed based on obtained darter CENP-A sequences either as optimum pairs or as individual primers that were compatible with relevant existing primers. Optimum annealing temperatures were determined by gradient PCR amplifications.

### 3.3. PCR Amplifications

Initial nested PCR amplifications of the Tallapoosa darter CENP-A HFD utilized the degenerate primer pairs S5L–A5L and S3s–A3s. The reaction mixture for the initial reaction was 25 μL of Qiagen HotStarTaq Master Mix, 100 ng of genomic DNA, 12 μM of each primer in a total volume of 50 μL. The PCR conditions were 95 °C for 15 minutes to activate the Taq polymerase followed by 35 cycles of 30 seconds at 94 °C, 1 minute at the annealing temperature (Table 1) and 3 minutes at 72 °C. The final cycle was followed by a 10 minute incubation at 72 °C. The conditions were the same for the nested reaction except that 1 μL of a 1 to 100 dilution of the gel purified (Zymoclean Gel DNA Recovery Kit, Zymo Research) primary PCR product was used instead of genomic DNA and the number of cycles was reduced to 20.

GenomeWalker PCR reactions were carried out on Tallapoosa darter restriction digested DNA libraries according to manufacturer's instructions utilizing the Clontech Genome Walker Universal kit and gene specific primers designed as part of this study (Tables 2 and 3). Initial walks utilized the Qiagen HotStarTaq Master Mix but subsequent walks utilized the Clontech Advantage 2 Polymerase PCR Kit. Where walks could not be extended with the initial kit produced restriction digested DNA libraries, additional libraries were constructed utilizing restriction enzymes BsaA1, MslI, MspA1I, and SspI.

All other PCR reactions utilized the conditions above except that non-degenerate primer concentrations were 0.5 μM and extension temperatures varied from 1 to 7 minutes depending on the anticipated length of the PCR product (1 minute per 1000 nucleotides). Annealing temperatures for various primers are listed in Table 4. For PCR amplification of *Palmoris* sequences, Advantage 2 Polymerase PCR Kit (Clontech) was utilized.

### 3.4. Cloning of PCR Products

Gel purified PCR products (Zymo Gel DNA Recovery Kit, Zymo Research) from nested PCR reactions and GenomeWalker PCR reactions were cloned into pSMART GC HK plasmids utilizing the Lucigen GC Cloning and Amplification Kit according to manufacturer's instructions.

### 3.5. DNA Sequencing and Sequence Analysis

All sequencing was performed by Functional Biosciences, Inc., Madison WI (functionalbio.com). Recombinant plasmids of cloned amplimers were isolated utilizing the Zippy Plasmid Miniprep Kit (Zymo Research). The fragments cloned in the pSMART plasmids were sequenced from both ends utilizing SL1 and SL2 primers (Lucigen). Where the size of the inserts exceeded the read lengths, new sequencing primers were designed based on the initial sequence reads and additional sequencing cycles and primer designs were carried out as necessary. Where necessary, additional primers were designed to obtain sequences of all exons from both strands.

Sequences were assembled into contigs and aligned with Geneious Pro bioinformatics software (Biomatters Ltd.).

### 3.6. Testing for Positive Selection

Pairs of sequences were initially tested for regions showing signs of positive selection (Ka/Ks > 1) utilizing sliding window analysis software SWAKK [18]. Statistical significance of positive selection was tested with the Z-test of Selection and with Fisher's Exact Test of Selection as implemented in MEGA 5 [25].

## 4. Conclusions

We have demonstrated that the Tallapoosa darter CENP-A gene HFD sequences can be isolated from genomic DNA by nested PCR in a manner that does not lead to the amplification of the highly sequence related histone H3 gene. We also demonstrated that PCR based walking can be subsequently used to isolate the rest of the fish CENP-A gene and adjacent gene sequences. With PCR primers anchored in these adjacent gene sequences, we were able to isolate CENP-A sequences from two *Percina* species. Comparisons of the three percid CENP-A genes shows that adaptive evolution has occurred in the N-terminal tail of this gene.
